# Dysregulation of the Gut-Adipose Tissue-Liver Axis: a Possible Mechanism Behind the Relationship Between Metabolic Dysfunction-Associated Steatotic Liver Disease and Type 2 Diabetes

**DOI:** 10.1007/s11892-026-01623-8

**Published:** 2026-04-11

**Authors:** Susanna Longo, Tommaso Giovanni Rinaldi, Jose Manuel Fernández-Real, Massimo Federici

**Affiliations:** 1 Department of Systems Medicine and Center for Atherosclerosis, University of Rome Tor Vergata, Via Montpellier 1, 00133 Rome, Italy; 2 Nutrition, Eumetabolism and Health Group, Girona Biomedical Research Institute (IDIBGI-CERCA), Girona, Spain; 3 Department of Diabetes, Endocrinology and Nutrition, Dr. Josep Trueta University Hospital, Girona, Spain; 4 Integrative Systems Medicine and Biology Group, Girona Biomedical Research Institute (IDIBGI-CERCA), Parc Hospitalari Martí I Julià, Edifici M2, Salt, Spain; 5 CIBER Fisiopatología de La Obesidad y Nutrición (CIBERobn), Instituto de Salud Carlos III, Madrid, Spain; 6 Department of Medical Sciences, School of Medicine, University of Girona, Girona, Spain; 7 IRCCS Neuromed, Pozzilli, IS Italy

**Keywords:** Gut-adipose tissue-liver axis, Gut microbiota, MASLD, Type 2 diabetes, Personalized microbiome-based therapies

## Abstract

**Purpose of Review:**

Metabolic dysfunction-associated steatotic liver disease (MASLD) is a hepatic manifestation of metabolic syndrome, frequently occurring alongside type 2 diabetes (T2D), and it can present in varied phenotypes. This review provides a critical analysis of gut-adipose tissue-liver axis (GALA) dysregulation in MASLD pathogenesis, contextualizing the discussion within both established and emerging paradigms. The review elucidates how GALA dysregulation shapes the interplay between MASLD and T2D, emphasizing inter-organ crosstalk among the gut, liver, and adipose tissue, and highlighting the role of microbial metabolites, notably bile acids. The review further summarizes recent advances in stratifying MASLD into distinct clusters, examining intricate associations with cardiometabolic comorbidities, and critically evaluates novel therapeutic approaches targeting GALA modulation.

**Recent Findings:**

MASLD can show heterogeneous phenotypes. It significantly increases the risk of developing new-onset T2D, and both conditions often coexist due to their shared pathophysiological basis in insulin resistance. The gut microbiota influences immune function and modulates host metabolism by regulating glucose tolerance and insulin sensitivity through a specific crosstalk between the gut, liver, and adipose tissue. The dysregulation of the GALA may be a mechanism underlying the interplay between MASLD and T2D, influencing IR and metabolic syndrome.

**Summary:**

A thorough investigation of GALA's role in the physiopathogenesis of MASLD and T2D highlights its potential to distinguish specific MASLD clusters and to identify personalized therapeutic strategies.

**Graphical Abstract:**

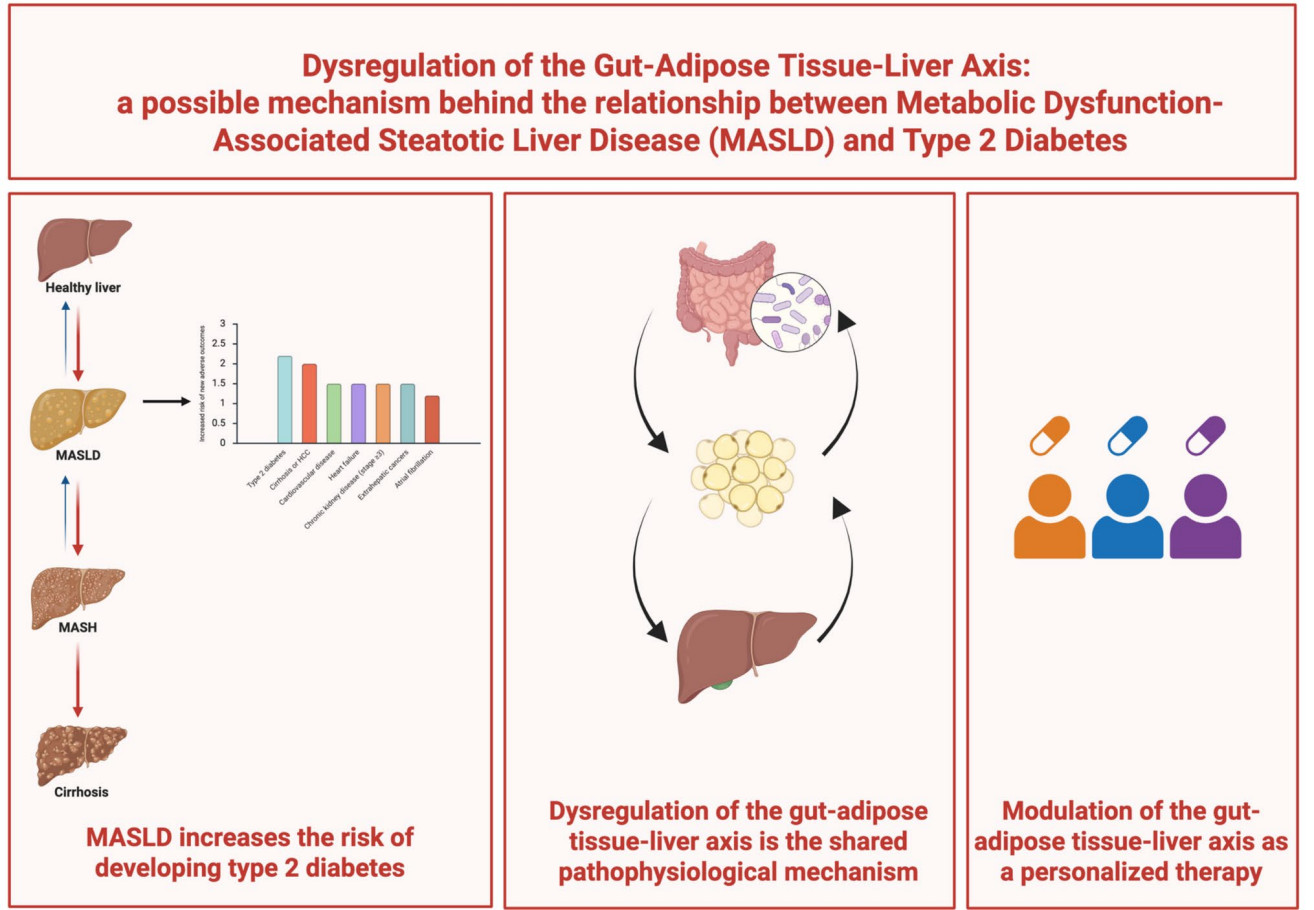

## Introduction

Metabolic dysfunction-associated steatotic liver disease (MASLD) is defined as the presence of excess triglyceride accumulation in the liver alongside at least one of the five hallmarks of metabolic syndrome (MetS) in the absence of clinically significant alcohol consumption and other secondary causes of steatosis. The spectrum of MASLD includes steatosis, metabolic dysfunction-associated steatohepatitis (MASH), fibrosis, cirrhosis, and MASH-related hepatocellular carcinoma (HCC) [1].

With its significant socioeconomic impact, MASLD poses a major global health challenge, adversely affecting health-related quality of life. It has become the most common chronic liver disease, impacting up to 38% of the adult population worldwide. Furthermore, the phenotypic presentation of MASLD can be significantly heterogeneous, and individuals with MASLD may exhibit diverse degrees of metabolic dysfunction and different risks of extrahepatic complications. Cardiovascular diseases (CVD) are the leading cause of death among individuals with MASLD, and MASLD raises the risk of developing new-onset type 2 diabetes (T2D) by 2.2 times [2]. The prevalence of MASLD among people with T2D is estimated to be at least 70%. The combination of MASLD and T2D significantly increases the risk of progression to MASH, cirrhosis, and HCC, as well as the overall risk of liver-related mortality [3].

The increasing incidence of MASLD, its heterogeneous presentation, and its strong association with T2D underscore the crucial need for a personalized therapeutic approach. To achieve this goal, it is essential to explore different pathological factors beyond traditional pathophysiological paradigms focused on established drivers. It is well-established that the relationship between T2D and MASLD is influenced by insulin resistance (IR) and visceral adiposity, which contribute to the onset and progression of both conditions [4]. Delving into new and promising prospects, the gut microbiota (GM) is emerging as a crucial link between liver dysfunction and impaired insulin secretion. GM influences immune function and modulates host metabolism by regulating glucose tolerance and insulin sensitivity through a specific crosstalk between the gut, liver, and adipose tissue (AT). It suggests that dysregulation of the gut-adipose tissue-liver (GALA) axis may be a mechanism underlying the relationship between MASLD and T2D, influencing IR and MetS [5]. Investigating these connections could enhance our understanding of diseases, helping to phenotype diverse clusters of MASLD and leading to the discovery of novel targets for personalized diagnosis and therapy [6].

By moving beyond traditional pathophysiological paradigms, this review provides a critical analysis of GALA dysregulation in MASLD pathogenesis. The review elucidates how GALA dysregulation shapes the interplay between MASLD and T2D, emphasizing inter-organ crosstalk among the gut, liver, and adipose tissue, and highlighting the role of microbial metabolites, notably bile acids (BA). The review further summarizes recent advances in stratifying MASLD into distinct clusters, examining intricate associations with cardiometabolic comorbidities, and critically evaluates novel therapeutic approaches targeting GALA modulation.

It is specified that the term MASLD will be used to refer to older definitions, such as non-alcoholic fatty liver disease (NAFLD) and fatty liver disease associated with metabolic dysfunction (MAFLD).

## The Crosstalk Between the Gut and the Liver

Gut–liver crosstalk is central to the conventional pathophysiology of MASLD. The liver, as a metabolic hub connected to the gut through the intestinal barrier and portal circulation, receives nutrient-rich blood from the gut and regulates glucose, lipid, and amino acid metabolism in response to gut signals, including microbial metabolites and incretin hormones like glucagon-like peptide-1 (GLP-1) and glucose-dependent insulinotropic peptide (GIP) [7]. These hormones are crucial for glucose regulation and IR, while GIP also enhances white AT storage. Changes in incretin levels and signaling, characterized by diminished GLP-1 activity and an obesity-induced downregulation of GIP receptors, are implicated in the pathogenesis of MASLD and MASH [5, 8].

Beyond established pathophysiological drivers of MASLD, such as IR and visceral AT dysfunction, causing ectopic fatty acid accumulation and liver overload, the GM plays a crucial role in gut–liver crosstalk and disease progression. The GM is a complex ecological system that spans the entire intestinal tract, and its composition and functions vary significantly depending on genetics, diet, and lifestyle [9]. GM is crucial for maintaining host physiological homeostasis and regulating immune responses [10, 11]. There is a constant interplay between the GM and the liver via portal circulation and the biliary tract. The GM contributes to various liver diseases, while the liver influences the prevalence and behavior of the bacteria [12].

**Fig. 1:**
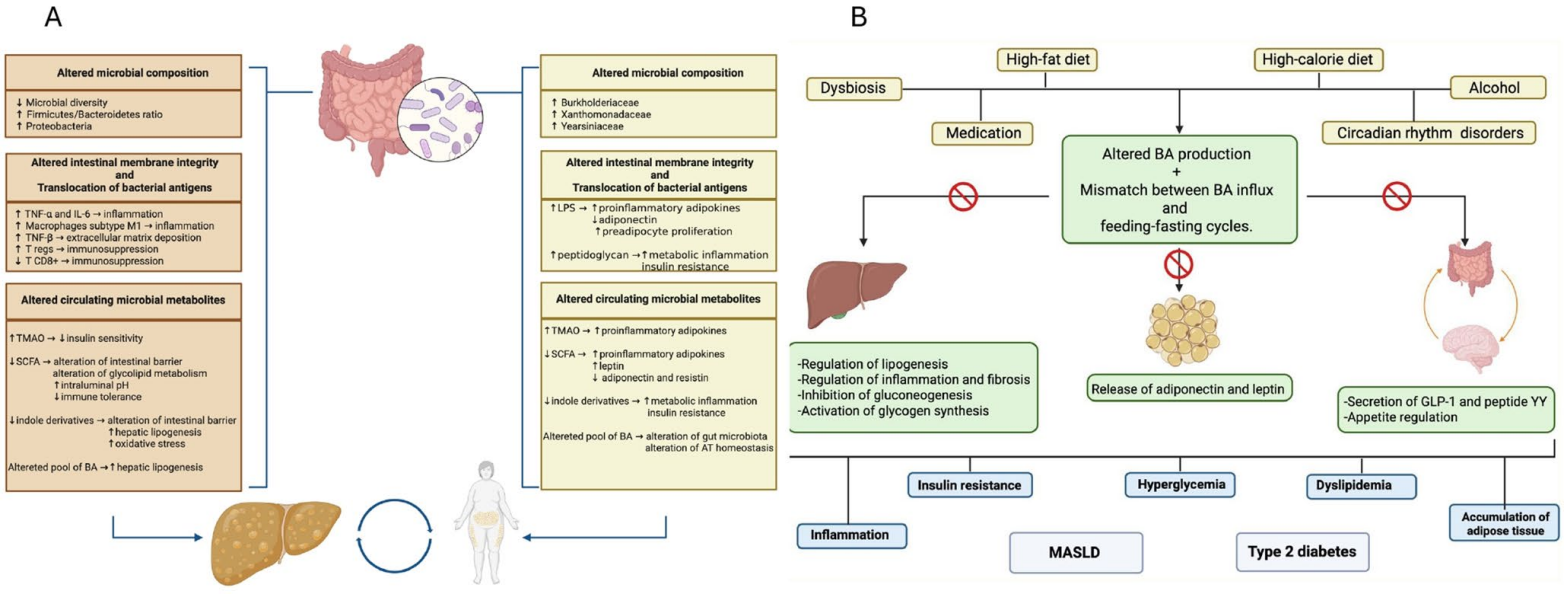
The Gut-Adipose Tissue-Liver Axis. Panel **A** illustrates the impact of altered gut microbiota composition, compromised intestinal barrier integrity, and microbial metabolites on the pathogenesis of hepatic steatosis (depicted on the left, in light brown) and the expansion of adipose tissue (depicted on the right, in light yellow). Panel **B** depicts the factors contributing to dysregulated bile acid synthesis and the disruption of synchrony between bile acid inflow and the fasting–feeding cycle, along with their subsequent metabolic consequences. AT: Adipose Tissue; BA: Bile Acid; GLP-1: Glucagon-Like Peptide-1; IL-6: Interleukin 6; LPS: Lipopolysaccharide; MASLD: Metabolic Dysfunction-Associated Steatotic Liver Disease; SCFA: Short-Chain Fatty Acid; T CD8 + : Cytotoxic T lymphocyte; T reg: Regulatory T cell; TMAO: Trimethylamine N-oxide; TNF-α: Tumor Necrosis Factor alpha; TNF-β: Tumor Necrosis Factor beta

Dysbiosis is linked to cardiometabolic diseases like obesity, T2D, and MASLD [13, 14] (Fig. 1A). Unhealthy diets and lifestyles alter GM composition, increasing the Firmicutes/Bacteroidetes ratio in MASLD [15]. Furthermore, progression from mild steatosis to advanced fibrosis is associated with increased Proteobacteria and decreased Firmicutes [16]. Astbury et al. reported reduced microbial diversity also in biopsy-proven MASH, particularly in cirrhosis [17]. Additionally, MASLD male mice exhibit lower microbial diversity and higher endotoxins and inflammatory mediators than females, suggesting sex-specific dysbiosis [18]. MASLD prevalence exhibits distinct age- and sex-specific patterns: incidence increases in men until middle age and declines after 50–60 years, whereas postmenopausal women demonstrate a higher incidence than age-matched men [19]. Alterations in GM composition, in conjunction with hormonal fluctuations, may further modulate these epidemiological patterns.The intestinal barrier is crucial for maintaining GM homeostasis, and its disruption contributes to MASLD. The intestinal barrier acts as a selective filter, regulating nutrient absorption and preventing microbial translocation [20]. Unhealthy diet, inflammation, drugs, alcohol, and dysbiosis impair intestinal barrier function, thereby increasing gut permeability [21]. Increased permeability allows microbial antigens, such as lipopolysaccharides (LPS), peptidoglycans, and bacterial DNA, to reach the liver, activating Toll-like receptor 4 (TLR4) signaling pathways on hepatic cells, and proinflammatory cytokine release, promoting MASLD progression [22]. Advanced fibrosis involves hepatic stellate cell activation and extracellular matrix deposition, mediated by TNF-β signaling, with inflammasome activation further promoting fibrogenesis [23–25]. TLR4-deficient mice are resistant to MASLD, highlighting the role of the LPS-TLR4 axis [26]. Increased intestinal permeability also shifts macrophage polarization from the anti-inflammatory M2 to the proinflammatory M1 phenotype, exacerbating hepatic lipid accumulation and IR. The effects of GM alterations on the hepatic immune system in MASLD-HCC have also been studied. Microbiome-derived molecules in MASLD-HCC promote immunosuppression by expanding T regulatory cells and reducing the T CD8 + cytotoxic response, facilitating progression to liver cancer [27].

GM influences MASLD progression through microbial metabolites, such as trimethylamine N-oxide (TMAO). Choline is converted to trimethylamine (TMA) by GM and oxidized to TMAO in the liver [28]. TMAO worsens MASLD by promoting IR, adipose inflammation, and disrupting BA–Farnesoid X Receptor (FXR) signaling [29].

TMAO has been implicated in the pathogenesis of major comorbidities associated with MASLD, including T2D, obesity, and hypertension, as evidenced by a recent meta-analysis [30]. Consequently, elevated circulating TMAO concentrations may facilitate the progression from MASLD to MASH, cirrhosis, and HCC, thereby increasing the overall risk of liver-related mortality. Moreover, TMAO may underlie the phenotypic heterogeneity observed in MASLD, as evidenced by a murine study in which circulating TMAO predicted the future onset of obesity and impaired glucose tolerance [31]. Short-chain fatty acids (SCFA) derived from microbial fermentation of undigested fibers protect against MASLD by supporting epithelial energy metabolism, barrier integrity, immunotolerance, and metabolic homeostasis [32, 33]. Butyrate protects against hepatic steatosis, and all SCFA are significantly reduced in stool samples from MASLD patients [34]. Restoring SCFA-producing bacteria or supplementing SCFA improves lipid metabolism and liver inflammation by enhancing the quality of the intestinal barrier and modulating inflammatory pathways [35]. GM metabolizes tryptophan into indole derivatives, such as indole-3-acetic acid (IAA) and indole-3-propionic acid (IPA), which protect against MASLD by inhibiting the nuclear factor kappa B (NF-kB) pathway and IL-8 release, thus preserving gut integrity [36]. IAA reduces the production of free fatty acids (FA) and hepatic inflammation [37]. In murine models, it mitigates MASLD by reducing hepatic lipogenesis and oxidative stress [38]. The amount of IAA-producing bacteria decreases in mice fed a high-fat diet (HFD), and indole derivatives are lower in fecal samples from both mice and humans with MASLD [39]. GM modulates MASLD development through BA. Primarily BA, synthesized in the liver from cholesterol, aids in lipid and fat-soluble vitamin absorption. After reabsorption via enterohepatic circulation, GM converts the remaining BA into secondary BA. BA are endogenous ligands to the G protein-coupled receptor 5 (TGR5) in the liver, AT, and muscle. BA also bind the FXR, which then regulates lipogenesis, inflammation, and fibrosis [40]. TGR5 controls hepatic de novo lipogenesis, VLDL, and plasma triglyceride turnover independently of FXR [41]. The interplay between BA and GM is bidirectional. BA regulates bacterial growth and differentiation, and bacteria regulate BA homeostasis and their interaction with FXR and TGR5, modulating lipid metabolism, immunity, and intestinal barrier integrity [42]. MASLD/MASH is characterized by altered BA profiles and downregulation of FXR and TGR5 signaling [43, 44].

Overall, these findings underscore the complexity of GM-liver interactions in the pathogenesis of MASLD/MASH, highlighting the need for further research in human subjects to validate hypotheses derived from animal models.

## The Crosstalk Between the Gut and the Adipose Tissue

Clarifying GALA's impact on the MASLD–T2D relationship requires examining its role in established disease mechanisms. AT stores triglycerides as an energy reservoir, accounting for about 10–30% of total body weight in healthy weight individuals. Obese people exhibit a higher body fat percentage, typically exceeding 30% in men and 42–44% in women, though these values may vary with age and the specific assessment methodology [45]. A critical dimension of obesity pertains to the distribution of AT. Notably, abdominal or visceral AT is a principal pathogenic driver of IR and plays a dominant role in the pathogenesis of both MASLD and T2D. There are two types of AT. White adipose tissue (WAT) serves as an insulin-regulated energy store and supports thermal insulation, mechanical protection, endocrine secretion of adipokines, and immune regulation through chemokine release [46]. Brown adipose tissue (BAT) comprises a small portion of total AT and is mainly found in the supraclavicular region. It burns energy and generates heat through Uncoupling Protein 1 (UCP-1). Prolonged cold exposure and β-adrenergic activation induce WAT browning, thereby increasing energy expenditure and helping maintain body temperature [47]. The traditional pathophysiological models illustrate how alterations in AT homeostasis are strongly linked to IR, T2D, and MASLD. IR, characterized by reduced insulin sensitivity and compensatory hyperinsulinemia, underlies T2D, its chronic complications, and systemic diseases like cancer, MASLD, and CVD [48–50]. Impaired glucose utilization related to IR leads to triglyceride storage and AT expansion; when AT capacity is exceeded, triglycerides accumulate ectopically in tissues such as the liver and muscle [51]. Both excess AT (as in obesity) and selective AT loss with ectopic lipid deposition (as in lipodystrophy) contribute to IR and MASLD [52, 53]. Hepatic triglyceride accumulation results in lipotoxicity, further worsening IR. In IR and obesity, AT expansion disrupts endocrine and immune functions by promoting a proinflammatory environment characterized by elevated reactive oxygen species (ROS) and proinflammatory adipokines [such as leptin, resistin, TNF-α, IL-6, plasminogen activator inhibitor 1 (PAI-1), and monocyte chemoattractant protein-1 (MCP-1)], alongside reduced anti-inflammatory mediators [such as adiponectin and peroxisome proliferator-activated receptor gamma (PPARγ)]. This imbalance underlies obesity-related IR and facilitates MASLD progression to MASH [46, 47].

Incretin hormones play a crucial role in the gut-AT communication and during the development of IR, MASLD, and T2D. After carbohydrate and triglyceride intake, GLP-1 and GIP promote insulin secretion, improve insulin sensitivity, and suppress appetite through their effects on the central nervous system, thereby limiting AT accumulation and supporting weight loss [54, 55]. GLP-1 stimulates insulin secretion directly and through vagus-mediated pathways. It also improves postprandial glucose regulation by delaying gastric emptying, increasing pyloric contractions, slowing the rate of glucose entry into the bloodstream, and enhancing satiety [5, 56]. GIP promotes triglyceride hydrolysis in AT and reduces free FA production; however, obesity-induced downregulation of GIP receptors impairs this effect [57, 58]. In obesity treatment, GLP-1 receptor analogues (GLP-1 RA) induce weight loss via central appetite suppression in both healthy individuals and patients with T2D [56]. Both agonism and antagonism of GIP receptors have been studied regarding body weight reduction. GIP receptor antagonism is less effective for weight loss in obesity, whereas combined GIP and GLP-1 receptor agonism improves glycemic control, suppresses appetite, and reduces body weight in T2D [58].

Exploring the impact of emerging potential factors in the pathogenesis of MASLD, recent evidence implicates GM and GALA in AT homeostasis, with their disruption promoting adiposity, obesity-related IR, and systemic inflammation [59] [Fig. 1A]. Obesity and MASLD are linked with changes in GM composition, with specific bacterial families enriched in obesity and MASLD, and distinct profiles between lean and obese MASLD patients [51]. Key bacterial families associated with obesity include *Burkholderiaceae*, *Xanthomonadaceae*, and *Yearsiniaceae*, whereas *Morganellaceae* is often absent in non-obese MASLD. Microbiome composition shifts may alter molecular pathways, such as adenosylcobalamin and chorismate, and increase energy extraction from food in obesity [60, 61].

GM cell membrane components, such as LPS, contribute to obesity-induced IR by promoting inflammation, reducing adiponectin production, and increasing adiposity [62]. Other key components of bacterial cell membranes include peptidoglycans, recognized by intracellular receptors NOD1 and NOD2. NOD1 activation and NOD2 deficiency are associated with IR and metabolic inflammation in HFD-treated mice [63, 64].

GM metabolites influence AT homeostasis. TMAO is associated with obesity and proinflammatory adipokine production via NF-κB and MAPK pathways [65]. GM imbalance impairs SCFA levels, diminishing their anti-inflammatory effects on adipokine and cytokine profiles [66, 67]. Indole derivatives modulate inflammation, insulin sensitivity, and fat accumulation [43]. Notably, IPA is reduced in obese individuals with low-grade IR-related inflammation [68]. Furthermore, research on mice subjected to an 8-week HFD followed by gastric bypass indicates that changes in BA production modulate GM composition and AT homeostasis [69]. GM-derived SCFA and BA also promote GLP-1 secretion and regulate WAT/BAT balance, WAT browning, and thermogenesis, which are influenced by microbial composition [5, 70–74], whereas LPS/TLR4/FOXC2 and TMA/FMO3/TMAO signaling hinder thermogenesis in obesity [75]. Alterations in microbial composition can reshape the spectrum of microbial metabolites, disrupting key biochemical pathways involved in host metabolism and disease progression.

Overall, GM regulates adiposity, IR, and inflammation, key mechanisms in MASLD/MASH and T2D. However, further exploration and validation through translational studies are essential to strengthen these associations and uncover their broader implications.

## The Crosstalk Between Adipose Tissue and the Liver

The liver–AT axis plays a central role in regulating metabolic responses to fasting and feeding, processes that become disrupted in metabolic diseases such as obesity, T2D, and MASLD. Their crosstalk is underpinned by the fact that both the liver and AT are key insulin-responsive organs, essential for coordinated glucose uptake and overall metabolic balance. In both the fasting and fed states, the liver and AT engage in bidirectional metabolic communication, primarily through lipoprotein exchange. During the fed state, AT releases free FA that are subsequently taken up by the liver. Conversely, the liver secretes lipoproteins, notably very low-density lipoproteins (VLDL), in response to insulin, which also promotes glycogenesis and de novo lipogenesis in hepatocytes. These VLDL particles facilitate the transport of triglycerides and cholesterol from the liver to AT. In IR, the lipoprotein profile shifts toward increased hepatic VLDL production, elevated levels of intermediate-density lipoproteins (IDL), arising from VLDL metabolism by lipoprotein lipase, reduced high-density lipoproteins (HDL) size and concentration, and increased small, dense low-density lipoproteins (LDL) particles. These changes result in the characteristic dyslipidemia seen in IR-related conditions such as obesity, T2D, and MASLD. In individuals with MASLD and expanded visceral AT due to obesity, persistent hyperinsulinemia resulting from IR stimulates hepatic de novo lipogenesis and augments the release of free FA from AT into the circulation. These processes collectively promote substantial intrahepatic triglyceride accumulation. Sustained hepatic lipid overload precipitates oxidative stress, which in turn promotes an environment conducive to hepatocyte injury and apoptosis, thereby advancing the progression of liver damage [76].

Lipotoxicity in MASLD extends beyond dysregulated lipoprotein metabolism, elevated free FA release from AT, and excessive hepatic triglyceride accumulation. Emerging in vivo evidence from non-diabetic individuals with MASLD implicates the activation of liver-resident macrophages or Kupffer cells as a consequence of AT dysfunction secondary to IR. The hepatic lipid deposition drives the pro-inflammatory polarization of Kupffer cells. Both activated Kupffer cells and infiltrating hepatic macrophages exacerbate hepatic IR and promote the progression in MASH [77]. Compelling evidence underscores the pivotal role of macrophage infiltration in the progression of MASLD to MASH. Both obesity and T2D are associated with elevated expression of growth and differentiation factor 15 (GDF-15) in AT, primarily due to macrophage accumulation, the key immune cell population responsible for GDF-15 synthesis in this context. Investigations on murine and human models of obesity and T2D-related MASH have consistently demonstrated a stepwise increase in circulating GDF-15 concentrations during the sequential progression from obesity, to T2D, and ultimately to MASH. Notably, as MASH develops, hepatocellular GDF-15 expression is further upregulated through stress-induced signaling pathways. Thus, the observed elevation in systemic GDF-15 reflects a dual contribution from both AT and hepatic tissues in the context of metabolic diseases, thereby identifying this increase as a promising therapeutic target for future interventions [78]. The intricate interplay between the liver and AT presents other potential avenues for therapeutic intervention. Emerging evidence indicates that WAT browning induction markedly elevates energy expenditure and enhances systemic metabolic homeostasis. This is evidenced by reductions in body weight, improvements in insulin sensitivity, and attenuation of hepatic steatosis and inflammation, particularly in the context of a high-fat diet. Preclinical studies in murine models further demonstrate that increased BAT activity augments thermogenesis, facilitates the catabolism of free FA, diminishes hepatic lipid accumulation and inflammation, and ameliorates liver fibrosis [79].

The dynamic interplay between AT and the liver represents a critical avenue for identifying novel therapeutic targets relevant to the pathogenesis of MASLD and its progression to MASH. Continued research elucidating these inter-organ mechanisms will be essential for guiding the development of targeted interventions.

## How Dysregulation of the Gut-Adipose Tissue-Liver Axis Affects the Relationship Between MASLD and T2D: the Bile Acid Hypothesis

Given the complex interplay among the GM, gut, AT, and the liver, it is evident that the GALA can play a crucial role in the development of the metabolic dysfunction underlying both MASLD and T2D. GALA dysregulation and altered systemic levels of microbial metabolites impair glucose tolerance and insulin sensitivity, contribute to AT accumulation and low-grade inflammation associated with IR, and disrupt gut hormone production and immune regulation [5, 80].

BA have received considerable interest as potential mediators of the effects of GALA dysregulation on the development of MASLD and T2D (Fig. 1B). BA help absorb, distribute, metabolize, and excrete nutrients and drugs, while also acting as signaling molecules that regulate lipogenesis, inflammation, and fibrosis. They also act as hormones that manage glucose metabolism alongside insulin and glucagon, following a circadian rhythm that aligns metabolic responses with nutrient availability during feeding and fasting. By activating FXR, BA inhibit gluconeogenesis and promote glycogen synthesis while influencing appetite by stimulating GLP-1 secretion [81]. Furthermore, BA can stimulate adiponectin and leptin release in AT, improving insulin sensitivity in HFD mouse models [82]. Conversely, insulin secretion influences the composition of the BA pool, affecting BA 12α-hydroxylase activity, which plays a crucial role in the development of IR [83]. A balanced composition of the BA pool, with the proper mix of primary and secondary BA, maintains metabolic balance and prevents hyperglycemia, dyslipidemia, and obesity [84]. Secondary BA-producing intestinal microbes play a key role in glucose homeostasis, lipid metabolism, and energy expenditure [85].

Given these physiological insights, it is reasonable to hypothesize a role of BA in mediating the mechanisms behind the coexistence of MASLD and T2D. Changes in BA concentrations and composition, as well as the desynchronization of systemic BA influx relative to the feeding-fasting cycle, may contribute to IR-associated obesity [86]. Dysbiosis, along with other factors such as HFD and high-calorie diets, alcohol, medications, and sleep or circadian rhythm disorders, can alter BA production, leading to MASLD, obesity, T2D, and CVD [84]. Nevertheless, the relationship between BA and GM is mutual, as BA regulate bacterial growth and differentiation. Consequently, dysregulation of the circadian rhythm of BA flow into the enterohepatic circulation may compromise the reciprocal interactions between BA and GM, worsening metabolic disorders and promoting diet-induced obesity [87]. Individuals with obesity and IR tend to have higher fasting BA levels but exhibit a modest increase in BA after eating, compared with individuals of normal weight. This observation suggests a disruption in the circadian rhythm governing BA production and release, along with alterations in BA-mediated metabolic pathways. This hypothesis is confirmed by studies on obese people who display abnormalities in BA transport, and experience reduced hepatic BA uptake and postprandial BA release [88]. Alterations in the circadian rhythm of postprandial BA plasma concentrations can negatively impact energy metabolism and appetite control through altered secretion of anorexigenic hormones, which in turn affects glycemic control, accumulation of AT, and subsequent IR [5].

Evidence from animal and human studies of obesity treated with bariatric surgery suggests GALA mediates MASLD–T2D interplay by regulating BA secretion and pooling (Table 1). Metabolic benefits post-surgery precede weight loss, implicating BA alterations and GM modulation beyond weight reduction [94, 95]. Bariatric surgery, especially Roux-en-Y gastric bypass, elevates serum BA levels by enhancing reabsorption in the terminal ileum and promoting conversion of primary to secondary BA in the colon [89]. Elevated secondary BA after surgery is associated with a shift in GM composition and enhances GLP-1 secretion, glucose tolerance, and hepatic outcomes, independent of weight loss [90, 91]. GM-modulated increases in secondary BA and a decreased primary-to-secondary BA ratio after bariatric surgery enhance incretin secretion, β-cell function, insulin sensitivity, and appetite regulation, independent of caloric restriction and weight loss [96]. These changes improve glycemic control in T2D, result in an 85% resolution rate of MASLD/MASH, and improve both histological and biochemical markers of MASLD and MetS [93, 96].

**Table 1:** The impact of bile acids on metabolic outcomes and their regulation based on gut microbiota’s changes

Models	Treatments	Results	Reference
Obese rats	Bile diversion and Sham surgery	In the BD group: Increased total and conjugated secondary bile acids in the serum;reduced weight and decreased levels of steatosis	[89]
Obese humans, rats and canine models	Roux-en-Y gastric bypass and gastric banding	In the RYGB group: Increased total plasma bile acids concentrations, FGF19, GLP-1, and PYY	[90]
Obese mice and lean controls	AIN‐93G diet for 12 weeks	In the obese group: Reduced faecal taurine‐conjugated BAs and butyrate‐producing bacteria; increased levels of steatosis	[91]
Obese humans	Laparoscopic sleeve gastrectomy	Decreased primary glycine and taurine-conjugated BAs, increased secondary BA and glycine-conjugated urodeoxycholic acid; reduced insulin resistance and proinflammatory cytokines	[92]
Obese humans	Bile diversion and Roux-en-Y gastric bypass	In both groups: Increased conjugated secondary BA; increased insulin sensitivity	[93]

*BD* Bile diversion; *RYGB* Roux-en-Y gastric bypass; *FGF19* Fibroblast growth factor 19; *GLP1* Glucagon-like peptide-1; *PYY* Peptide YY; *BA* Bile acids

Targeting plasma BA and GM-mediated BA conversion has therapeutic potential to improve insulin sensitivity in T2D and MASLD. However, additional research is required to assess the efficacy of microbiome-based treatments and their metabolites.

## Role of Gut Microbiota in Clustering MASLD Phenotypes

MASLD exhibits marked interindividual variability in progression, extrahepatic outcomes, and therapeutic response, complicating diagnosis, and management. Ongoing research aims to define MASLD clusters based on pathophysiology and comorbidities. The integration of omics techniques offers promise for identifying phenotype-specific molecular signatures [97–99].

Raverdy et al. [6] identified two principal MASLD phenotypes, cardiometabolic and liver-specific, using a clustering approach incorporating clinical, histological, genetic, transcriptomic, and metabolic features and extrahepatic complication risk factors. They exhibited similar hepatic histological and MRI characteristics, and both clusters were associated with an increased risk of chronic liver disease. However, the cardiometabolic cluster demonstrated increased incidence of CVD and T2D. In contrast, the liver-specific cluster exhibited earlier and more pronounced hepatic enzyme elevations, accelerated liver disease progression, and lower CVD and T2D risk. The liver-specific cluster showed greater enrichment of genetic variants used to calculate the polygenic risk score for liver fat content (PRS-HFC) compared to the cardiometabolic cluster. This trend was also evident when focusing solely on the PNPLA3 rs738409 variant, which, along with the TM6SF2, MBOAT7, and GCKR variants, is linked to hepatic fat accumulation, rapid disease progression, and advanced liver outcomes [100]. Transcriptomic analysis showed upregulation of cholesterol biosynthesis and glycolysis genes in the cardiometabolic cluster, indicating heightened metabolic dysfunction and elevated T2D and CVD risk. In contrast, genes involved in lipid homeostasis and inflammation were upregulated in the liver-specific cluster, corresponding to increased liver enzyme levels. Metabolomics showed elevated GM-derived metabolite levels in the cardiometabolic cluster, including tyramine O-sulfate, homocitrulline, p-cresol glucuronide, phenylacetylglutamine, phenylacetylglutamate, 4-hydroxyphenylacetylglutamine, 4-hydroxyphenylacetate, and imidazole propionate. These metabolites, derived from aromatic amino acids, are linked to IR, diabetes, and CVD risk [101], which may partly explain the increased CVD and T2D risk observed in this cluster. Intriguingly, deoxycholate, a secondary BA, was also elevated in the cardiometabolic cluster, implicating disrupted lipid metabolism. In contrast, the liver-specific cluster was characterized by hepatocyte-restricted alterations in lipid metabolism, consistent with its genetic profile.

Raverdy et al. [6] investigated molecular differences between the cardiometabolic cluster and the T2D control group, given the high prevalence of T2D in this cluster. Liver transcriptomics revealed a distinct signature in the cardiometabolic cluster, with overexpression of genes involved in metabolic and inflammatory pathways compared with T2D controls. Despite some overlap, the cardiometabolic cluster exhibited a distinct metabolomic profile, including unique BA metabolites linked to dysregulated lipid, protein, and energy metabolism, inflammation, and GM interactions.

The identification of two MASLD clusters underscores the necessity for personalized therapeutic approaches. MASLD phenotypic heterogeneity may explain therapeutic failures in clinical trials. The liver-specific cluster may benefit from Resmetirom, which reduces hepatic lipid content and liver inflammation. In contrast, cardiometabolic cluster may respond better to agents targeting metabolic dysfunction, such as GLP-1RA, both as monotherapy and in combination with GIP or glucagon agonists [99]. Raverdy et al. [6] provide a foundation for investigating the role of GM and its metabolites in MASLD phenotyping and precision medicine. Given the central pathogenic role of visceral obesity, future research should delineate a MASLD cluster characterized by visceral adiposity. Such efforts would enable a more precise elucidation of its direct contribution to MASH progression and facilitate the assessment of differential therapeutic responses within this subgroup.

## Therapeutic Targets in MASLD

### Current Recommendations and Recent Evidence

Lifestyle modification, including substantial weight loss achieved through regular exercise and calorie restriction, remains central for the management of MASLD/MASH and T2D, though long-term adherence is challenging. Pharmacologic options are limited, with Resmetirom recently approved for non-cirrhotic MASH and moderate to advanced fibrosis [2].

Some antidiabetic medications, including pioglitazone and GLP-1RA, are recommended for T2D patients with MASH or at high risk of advanced liver fibrosis [4, 102]. In phase 2 and 3 studies, Semaglutide and Efruxifermin (a bivalently anchored analogue of fibroblast growth factor 21) have shown efficacy in improving fibrosis in advanced MASH with T2D or MetS [103]. In phase 2 studies, Tirzepatide and Turvodutide (dual agonists targeting GLP-1 and GIP or glucagon receptors, respectively) show promise for MASH resolution, with efficacy similar to that of bariatric surgery [104, 105, 102]. Nonetheless, the relative contributions of weight loss and direct Tirzepatide effects to hepatic improvement are undetermined [106, 103].

Sodium-glucose co-transporter 2 inhibitors (SGLT2i) exhibit hepatoprotective effects in MASLD with or without T2D [107, 108]. Additional agents, such as low-dose aspirin, are under investigation for their potential to reduce hepatic steatosis [109].

### Future Perspective: Gut Microbiota as a Novel Therapeutic Target in MASLD

MASLD management must consider phenotypic heterogeneity and variable cardiometabolic risk, highlighting the need for novel therapeutic targets [110]. GALA and GM modulation represent promising personalized therapeutic approaches (Table 2).

**Table 2:** The impact of gut microbiota modulation on hepatic and metabolic outcomes

Classification	Compounds	Models	Treatments	Results	Reference
Prebiotics	A blend of peanut peel, geniposide, and isoquercitrin	Mice with MASLD	80 mg/kg PSE, 50 mg/kg GEN, or 5 mg/kg IQ for 12 weeks	Reduced body weight, improved liver function and steatosis	[111]
	Cilostazol	Mice with MASLD	Cilostazol 30,60 or 120 mg/kg for 12 weeks	Improved gut microbiota diversity, inhibited hepatic lipogenesis and gluconeogenesis, reduced steatosis, and inflammation	[112]
	Iron	Pathogen-free mice vs germ-free mice, on normal or low-iron diet	350 mg/kg iron or 4–8 mg/kg iron for 10 weeks	MASLD development depended on presence of gut microbiota; germ free mice were protected from steatosis seen in pathogen-free mice	[113]
	Histidine	Human hepatocytes, mice, and Drosophila	In mice: a combination of histidine, serine, carnosine, and cysteine for 4 weeks; in Drosophilas: 8 g of histidine/L; in hepatocytes 500 µM of histidine for 48 h	Improved hepatic function and reduced de novo lipogenesis	[114]
	Theabrownin extracted from Qingzhuan tea	High fat diet-fed mice	QTB 180 and 360 mg/kg for 8 weeks	Reduced body weight and lipid accumulation, increased SCFA-producing bacteria, improved intestinal barrier and reduced steatosis	[115]
	A blend of Opuntia ficus indica, Theobroma cacao, and Acheta domesticus	Mice with MASLD and overweight humans	10 g of dehydrated nopal, cocoa powder and dehydrated cricket for 6 weeks	Enriched SCFA-producing microbiota in mice and humans, reduced hepatic steatosis in mice, decreased fat intake in humans	[116]
	Fibers	Humans with MASLD	Two-month fiber-enriched diet (6 g or 12 g)	Increased microbial diversity and SCFA production, improved metabolic profile	[117]
	Oat beta-glucan	Mice with MASLD	1 g of beta-glucan/kg for 24 weeks	Reduced hepatic inflammation and fibrosis progression	[118]
	Lupeol	Mice with MASLD	Lupeol 20,40 or 80 mg/kg for 8 weeks	Improved bile acid metabolism, reduced body weight, cholesterol, and hepatic steatosis, restored gut barrier	[119]
Probiotics	*Lactobacillus plantarum (LPJZ-658*)	Mice with MASLD	10^9^ cells of L. plantarum for 9 weeks	Maintained gut barrier integrity and improved systemic metabolism, reduced hepatic lipid accumulation and inflammation	[120]
	*Lactobacillus plantarum (LPJZ-658*)	Copper-deficient and high-sugar diet fed mice	10^9^ cells of L. plantarum for 6 weeks	Improved liver function and steatosis in copper deficient MASLD model, restored gut microbial composition and metabolic readouts	[121]
]	*Akkermansia muciniphila*	Obese and type 2 diabetic mice	2.10^8^ cfu of A.Muciniphila/0.2 mL for 4 weeks	Reduced body weight, improved glucose, and lipid metabolism	[122]
	*Kineothrix alysoides*	Mice fed normal diet and high-fructose/high-fat diet	10^10^ cfu of K.alysoides/200 μL for 10 weeks	Reduced weight gain and steatosis, improved lipid metabolism and gut composition	[123]
	*Phocaeicola vulgatus*	Mice with MASLD	10^9^ cfu of P.vulgatus/100 μL for 6 weeks	Attenuated steatosis progression and improved hepatic outcomes	[124]
Postbiotics	Acetate	Mice exposed to MC-LR	2 g of acetate/kg for 6 weeks	Reduced liver inflammation and cytokine release, restored acetate-producing bacteria	[125]
	IPA	Humans with low and high IPA levels, high fat diet-fed mice	20 mg of IPA/Kg for 4 days	Low circulating IPA associated with insulin resistance, overweight and features of MetS; IPA correlated inversely with markers of metabolic dysfunction	[126]
	IPA and IAA	Humans with and without MASLD, Western diet-fed mice	0.1 mg of IPA and IAA/ml for 12 weeks	Improved insulin sensitivity, reduced hepatic inflammation, decreased lipogenesis, and attenuated/prevented MASLD	[127]

*PSE* Peanut peel; *GEN* Geniposide; *IQ* Isoquercitrin; *QTB* Qingzhuan tea; *SCFA* Short-chain fatty acids; *MC-LR* Cyanotoxin Microcystin-LR; *IPA* Indole-3-propionic acid; *IAA* Indole-3-acetic acid; *MASLD* Metabolic dysfunction-associated steatotic liver disease; *MetS* Metabolic syndrome

Some prebiotics improve hepatic and metabolic outcomes. In murine MASLD models, blends of peanut peel, geniposide, and isoquercitrin reduced body weight and ameliorated steatosis and dysbiosis [111]. Cilostazol modulates the GM, enriching Akkermansia and improving glycolipid metabolism in mice with MASLD [112]. Dietary iron deficiency can lead to MASLD and MetS due to liver triglyceride accumulation. Changes in GM composition can mediate the impact of dietary iron deficiency on MASLD risk and MetS [113]. Histidine supplementation, mediated by GM activity, improves hepatic outcomes and reduces lipogenesis in animal models [114]. Qingzhuan tea brownin reduces body weight and improves lipid profiles by increasing SCFA-producing bacteria in HFD-induced obese mice [115]. A blend of Opuntia ficus indica, Theobroma cacao, and Acheta domesticus enriches SCFA-producing microbes, reducing hepatic steatosis in mice and fat intake in overweight individuals [116]. Dietary fibers modulate SCFA production and microbiota diversity, offering therapeutic potential for MASLD [117]. Oat beta-glucan exhibits hepatoprotective and anti-inflammatory effects in mice with MASLD, mediated by enrichment of SCFA-producing bacteria, such as *Ruminococcus* and *Lactobacillus* [118]. Lupeol enhances intestinal immunity via FXR signaling, reducing body weight, cholesterol, and hepatic steatosis in MASLD murine models [119].

Probiotic modulation of GM composition shows therapeutic promise in MASLD. *Lactobacillaceae* support hepatic health and reduce IR and lipid accumulation in the liver and ectopic tissues [128]. In particular, *Lactobacillus plantarum (LPJZ-658)* mitigates the effects of obesogenic diets in mice [120]. In murine MASLD models with hepatic copper deficiency, *LPJZ-658* improved liver function and steatosis, counteracting the deficiency, and GM composition [121]. *Akkermansia muciniphila* is linked to reduced body weight and improved metabolic outcomes both in humans and mice [122]. *Kineothrix alysoides* reduces weight gain and steatosis and improves lipid metabolism in high-fructose dietary murine models [123]. *Phocaeicola vulgatus* supplementation attenuates MASLD by modulating lipid metabolism [124].

Postbiotic supplementation may ameliorate metabolic dysfunction in MetS. In murine models, acetate administration attenuates microcystin-induced liver inflammation by modulating GM [125]. Short-term IPA treatment improves IR in dysbiosis [126]. IPA and IAA, GM-derived metabolites, regulate hepatic inflammation and lipid metabolism, mitigating MASLD in animal models [127].

These findings identify potential therapeutic targets for modulating GM and GALA; however, validation in human clinical trials remains necessary. Future research should investigate BA restoration and combined therapies that address distinct clusters of MASLD.

## Conclusion

MASLD is a heterogeneous multisystem disorder that increases cardiometabolic risk, including a 2.2-fold increase in incident T2D. It underscores the need for improved diagnostics and personalized therapies. The GALA dysregulation, including altered BA homeostasis, is central to MASLD–T2D pathogenesis. Integrating nutritional, pharmacological, and GALA-targeted interventions holds therapeutic promise, especially for individuals with coexisting T2D and obesity.

## Key References

- Cusi K, Abdelmalek MF, Apovian CM, Balapattabi K, Bannuru RR, Barb D, et al. Metabolic Dysfunction–Associated Steatotic Liver Disease (MASLD) in People With Diabetes: The Need for Screening and Early Intervention. A Consensus Report of the American Diabetes Association. Diabetes Care. 2025;48:1057–82. 10.2337/dci24-0094
  - ○ MASLD is a common but underdiagnosed complication of type 2 diabetes, particularly with obesity. Liver health remains underemphasized in diabetes care, highlighting the need for greater awareness and integration of hepatic assessment into diabetes management.
- Gabbia D, De Martin S. Targeting the Adipose Tissue–Liver–Gut Microbiota Crosstalk to Cure MASLD. Biology. 2023;12:1471. 10.3390/biology12121471
  - ○ Dysfunction of the gut microbiota contributes to MASLD by altering metabolic pathways in the gut, liver, and adipose tissue. Modulating the gut microbiota may impact the pathophysiological interactions among these organs.
- Raverdy V, Tavaglione F, Chatelain E, Lassailly G, De Vincentis A, Vespasiani-Gentilucci U, et al. Data-driven cluster analysis identifies distinct types of metabolic dysfunction-associated steatotic liver disease. Nat Med. 2024;30:3624–33. 10.1038/s41591-024-03283-1
  - ○ MASLD comprises two clinically relevant phenotypes with similar liver phenotypes but distinct biological profiles and clinical outcomes. Distinguishing these clusters is crucial for advancing personalized therapies.

## Data Availability

No datasets were generated or analyzed for this study. All figures and tables presented in this manuscript are original works produced by the authors. No previously published material has been utilized; consequently, permission for reproduction is not required.
